# Targeting RSF1 with tanshinone I inhibits cervical cancer cell growth through modulation of NF-κB pathway

**DOI:** 10.3389/fphar.2026.1855194

**Published:** 2026-08-12

**Authors:** Yuan Shen, Shun Lu, Qianna Zhao, Tongtong Cui, Hongqian Cui, Zhenwu Cui, Linlin Shan, Chao Guo, Lei Shi

**Affiliations:** 1 School of Pharmacy, Henan Medical University, Xinxiang, China; 2 School of Basic Medical Sciences, Henan Medical University, Xinxiang, China

**Keywords:** carboplatin, cervical cancer, NF-κB pathway, Rsf1, tan I

## Abstract

Cervical cancer ranks as the fourth most prevalent malignancy among women across the world. Tanshinone I (Tan I), a principal bioactive component of *Salvia miltiorrhiza,* has demonstrated broad therapeutic potential in various cancers, yet its mechanism of action against cervical cancer remains unclear. In this study, we demonstrate that Tan I exerts potent anti-cervical cancer effects by inhibiting proliferation and inducing apoptosis in SiHa and HeLa cervical cancer cell lines. Mechanistically, Tan I may target RSF1, as evidenced by the finding that overexpression of RSF1 attenuated Tan I’s inhibitory effect, whereas RSF1 knockdown enhanced its efficacy. Through RSF1 targeting, Tan I suppresses the NF-κB signaling pathway and downregulates key anti-apoptotic genes, including *XIAP*, *CFLAR*, *BCL2*, and *BIRC2*. Moreover, Tan I enhances the cytotoxic activity of carboplatin when used in combination. Together, these results indicate that Tan I inhibits cervical cancer cell growth by targeting RSF1 and suppressing the NF-κB pathway, supporting its further development as a promising therapeutic candidate against cervical cancer.

## Introduction

Cervical cancer is one of the most common malignancies affecting women worldwide. According to 2020 global cancer data, over 600,000 cases of cervical cancer were reported, with more than 300,000 deaths annually (Arbyn et al., 2020). Immunosuppression and infection with high-risk human papillomaviruses (HPVs) are closely related to the etiology of cervical cancer. Approximately 70% of cervical cancer cases globally are linked to HPV16 and HPV18. While conventional chemotherapeutic approaches are widely used, they are frequently associated with significant toxicity and the development of drug resistance. Consequently, natural compounds have emerged as promising therapeutic alternatives due to their potential efficacy and favorable safety profiles.

Danshen (the root and rhizome of *Salvia miltiorrhiza* Bge.) is a common traditional medicine in China and several other Asian countries. Danshen has long been used to enhance blood circulation, remove blood stasis, regulate menstruation, and relieve pain. Nowadays, it is frequently utilized in the treatment of cardiovascular diseases, inflammation, early cirrhosis, and tumors (Wang et al., 2020). Tanshinones, the primary lipophilic components of Danshen, exhibit anti-cancer activities against various common malignant tumors (Chen et al., 2014). The fat-soluble ingredients of tanshinones include crytotanshinone, dihydrotanshinone I, tanshinone I (Tan I), and tanshinone IIA (Tan IIA), all of which are highly effective in combating tumors. *In vitro* studies have shown that crytotanshinone suppresses the growth of breast carcinoma and hepatocarcinoma cells (Jin et al., 2022; Lee et al., 2012). Dihydrotanshinone I inhibits the progression of hepatocellular carcinoma, colon carcinoma, breast cancer, osteosarcoma, ovarian carcinoma, and human glioma cells (Chen et al., 2017; Jiang et al., 2022; Sun et al., 2022; Tan et al., 2020; Wang et al., 2022; Zhao et al., 2022). Tan IIA prevents the growth of gastric cancer, nasopharyngeal carcinoma, hepatocellular carcinoma, colorectal cancer, and cervix carcinoma stem cells (Guan et al., 2020; Qian et al., 2023; Qin et al., 2018; Ren et al., 2017; Wang et al., 2021). Tan I has also been claimed to possess anti-tumor properties in various tumors. For instance, Tan I suppressed the proliferation and mobility of malignant bone tumors by regulating JAK/STAT3 and Circ_0000376/miR-432–5p/BCL2 signaling pathways (Wang et al., 2019; Ye et al., 2022). Tan I suppressed leukemia cell proliferation by blocking the activity of EZH and upregulating EZH2 target genes (Huang et al., 2021). Additionally, Tan I promotes ferroptosis and pyroptosis in cisplatin-resistant gastric cancer cells by regulating KDM4D/p53 and NF- kappaB/Caspase-3 (8)/GSDME pathway (Wang et al., 2024; Xia et al., 2023). Tan I also induces apoptosis and regulates autophagy in ovarian cancer, cervical cancer, breast carcinoma, and hepatic carcinoma cells (Cui et al., 2022; Zheng et al., 2020; Zhou et al., 2020). Although Tan I’s anti-cancer potential has been demonstrated, the underlying molecular mechanisms-particularly the molecular targets of Tan I- have not been well elucidated.

Remodeling and spacing factor 1 (RSF1), located within the chromosome 11q13.5 locus, has been identified as a chromatin remodeling factor. RSF1 forms an ATP-dependent chromatin remodeling complex by interacting with human sucrose nonfermenting protein two homolog protein (hSNF2H). hSNF2H acts as the nucleosome-remodeling ATPase in this complex, while RSF1 functions as the histone chaperone. This complex is essential for nucleosome repositioning, which regulates transcription, DNA replication, and genome stability. RSF1 has been reported to be overexpressed in multiple types of cancers, including lung, colon, ovarian, breast, nasopharyngeal, gastric, rectal, and bladder cancer. Elevated levels of RSF1 are closely associated with tumor aggressiveness, patient outcomes, and chemoresistance. For instance, RSF1 overexpression promotes the malignant behavior of non-small cell lung cancer (NSCLC) cells through the regulation of cyclinD1 and ERK signaling (Li et al., 2012). RSF1 levels also affect the sensitivity of ovarian cancer and NSCLC cells to paclitaxel by modulating the NF-kappaB (NF-κB) pathway (Chen et al., 2018; Yang et al., 2014). Additionally, RSF1 contributes to cisplatin resistance in nasopharyngeal cancer cells by modulating the Ras-MAPK pathway (Liu et al., 2017). In this study, we demonstrated that Tan I exerts potent anti-proliferative and pro-apoptotic effects in cervical cancer cells. Mechanistic investigations revealed that Tan I interacts with RSF1, thereby disrupting the RSF1/NF-κB signaling axis. Notably, Tan I enhanced the antitumor activity of carboplatin when used in combination. Our findings suggest that Tan I suppresses cervical cancer cell proliferation through an RSF1-dependent mechanism, offering novel insights into its molecular targets and therapeutic potential for cervical cancer treatment.

## Materials and methods

### Materials

The apoptosis detection kit was purchased from KeyGen Biotech (KGA108, China). CCK-8 solution was obtained from Glpbio (GK10001, United States). RNAiso Plus reagent was sourced from Takara (9108, Japan). Reverse transcription kit and SYBR Green qPCR Premix were supplied by Jacksenbio (MR0502-1 and MS0601, China). Lipofectamine 3000 was purchased from Invitrogen (L3000008, United States), and RNA TransMate was obtained from Songon Biotech (E607402, China). Tan I (purity ≥97%) and carboplatin (purity ≥98%) were purchased from MACKLIN (C805203 and T815956, China). Bay 11–7082 was purchased from Beyotime (S1523, China), TNF-α from T&L Biotech (GMP-TL303-0100, China), and DARTS assay kit from Beyotime (S3206, China). Crystal violet staining solution was purchased from Genview (DC079, United States). For experimental treatments, dimethyl sulfoxide (DMSO) served as both vehicle control and solvent for Tan I and Bay 11–7082, with the final concentration not exceeding 0.1% (v/v). Tan I stock solutions were prepared in amber tubes and stored at −20 °C wrapped in aluminum foil, with all dilutions similarly protected from light. TNF-α was reconstituted in ddH_2_O at a concentration of 100 μg/mL. Carboplatin was prepared as a 10 mM aqueous stock solution immediately prior to use.

### Cell culture

The human cervical cancer cell lines HeLa and SiHa were sourced from ATCC. All cell lines were authenticated by sourced and short tandem repeat profiling and tested negative for *Mycoplasma* contamination. Cells were maintained in Dulbecco’s Modified Eagle Medium (DMEM) supplemented with 10% fetal bovine serum (FBS) at 37 °C in a humidified atmosphere containing 5% CO2.

### Cell transfection

The plasmid vectors were transfected into cervical cancer cells by using Lipofectamine 3000, and siRNAs were transfected into cells using RNA TransMate reagent according to the manufacturer’s description. The sequences of siRNA-RSF1 and siRNA-NC were 5ʹ-GGA​GAA​GAA​GGG​CUA​UCU​UTT-3ʹ and 5ʹ-UUC​UCC​GAA​CGU​GUC​ACG​UTT-3ʹ, respectively. The PLVX-Flag-RSF1 expression vector and empty vector control plasmid were obtained from Prof. Hengbin Wang (Massy Cancer Center, Virginia Commonwealth University).

### Cell proliferation assay

HeLa and SiHa cells were inoculated in 96-well plates at a density of 2,000 cells/100 μL/well. For time-dependent experiments, cells were treated with Tan I (6 μM) for 0, 24, 48, and 72 h. For dose-dependent experiments, cells were subjected to different doses of Tan I (0, 1.5, 3, 6, and 12 μM) for 48 h. The treated cells were incubated with the CCK8 solution at 37 °C condition for an additional 2 h. The cellular densities were determined using a microplate reader (Molecular Devices, SpectraMaxi3, United States) at 450 nm. This experiment was independently repeated three times.

### Colony formation assay

The cervical cancer cells were inoculated into 6-well plates with 1,000 cells/well. The next day, the cells were exposed to different concentrations of Tan I (0, 1.5, 3, 6, and 12 μM) for 12 days. Then the cell colonies were fixed using 4% paraformaldehyde and stained with 0.1% crystal violet solution. Finally, photographs were taken and the cell colonies with more than 50 cells were counted. This experiment was conducted independently three times.

### Apoptosis analysis

Cervical cancer cells were seeded into the 6-well plates with 4 × 10^5^ cells/well and treated with the indicated concentrations of Tan I (0, 6, 12 μM). After 48 h, the cells were double stained with Annexin V-FITC and PI, and analyzed the fluorescence intensity by flow cytometry (Invitrogen, Attune™ NxT, United States). This experiment was independently repeated three times.

### Western blotting assay

Western blotting was performed as previously described (Shi et al., 2022). To determine whether Tan I inhibited cell proliferation through NF-κB signaling pathway, cervical cancer cells were pretreated with vehicle (DMSO, control) or Bay 11–7082 (the NF-κB inhibitor, 1 μM) for 60 min. Following pretreatment, the cells were stimulated with either vehicle (DMSO, control) or Tan I (6 μM) for 48 h in combination with pretreatments (DMSO or Bay 11–7082). The primary antibodies involved in this study: RSF1 (Abcam, ab109002, UK), GAPDH (Proteintech, 60004-1-Ig, China), Cleaved-caspase9 (Cell signaling technology, 9509, United States), Caspase 9 (Cell signaling technology, 9508, United States), Cleaved-Caspase 3 (Proteintech, 68773-1-Ig, China), Caspase 3 (Proteintech, 68773-1-Ap, China), Bax (Affinity, AF0120, United States), Bcl-2 (Affinity, AF6139, United States), p-p65 (Cell Signaling Technology, 3031, United States), p65 (Cell Signaling Technology, 8242, United States), p-IκBα (Cell Signaling Technology, 9246, United States), p53 (Proteintech, 60283-2-Ig, China), IκBα (Cell Signaling Technology, 9242, United States).

### Quantitative real-time PCR

RNAiso Plus reagent was used in the extraction of cervical cancer cell total RNA. The 1 μg total RNA was reverse transcribed by using All-in-One Script RTpremix Kit. Real-time PCR was performed using SYBR Green qPCR Premix on the platform (Roche, LightCycler® 480 II, United States). The primers used in this study included: Bcl-2 (forward, 5′-GGT​CAT​GTG​TGT​GGA​GAG​CG-3′; reverse, 5′-AAG​CCA​GCC​TCC​GTT​ATC​CT-3′), BIRC2 (forward, 5′-TCC​AGC​CTT​TCT​CCA​AAC​CC-3′; reverse, 5′-ACC​AGC​TCT​TGC​CAA​TTC​TGA-3′), CFLAR (forward, 5′-TCA​AGG​AGC​AGG​GAC​AAG​TTA-3′; reverse, 5′-GAC​AAT​GGG​CAT​AGG​GTG​TTA​TC-3′), XIAP (forward, 5′-CAG​AGC​GGA​GTT​GGC​ATT​TC-3′; reverse, 5′-CTT​GTC​CAC​CTT​TTC​GCG​CC-3′), β-Actin (forward, 5′-AAG​GAG​CCC​CAC​GAG​AAA​AAT-3′; reverse, 5′-ACC​GAA​CTT​GCA​TTG​ATT​CCA​G-3′).

### Cellular thermal shift assay (CETSA)

CETSA assay was carried out as previously described with some minor adjustments (Lu et al., 2024). In brief, the cervical cancer cells were subjected to 6 μM Tan I or no treatment for 12 h. Then the cells were washed and harvested with PBS solution containing proteinase inhibitor. The collected cells were separated into eight tubes (100 μL/tube), and each tube was heated for 3 min at 41, 45, 49, 53, 57, 61, 65, and 69 °C. The above samples were repeatedly freeze-thawed three times with liquid nitrogen, clarified at 12,000 rpm at 4 °C for 15 min, and then analyzed by Western blotting.

### Drug Affinity Responsive Target Stability (DARTS)

The Drug Affinity Responsive Target Stability (DARTS) assay was performed using the Beyotime DARTS Assay Kit (S3206) according to the manufacturer’s instructions with minor modifications. Cervical cancer cell lysates were prepared using the provided Lysis Buffer supplemented with protease inhibitors. Cell lysates (100 μg total protein in 100 μL) were incubated with Tan I (6 μM) or an equal volume of vehicle control (DMSO) at room temperature for 60 min with gentle shaking. Following incubation, the samples were treated with varying concentrations of Pronase (0, 0.02%, 0.05%, 0.1%, 0.2%) and incubated at 40 °C for 10 min. The digestion was terminated by adding 1 μL of Protease Inhibitor Cocktail (100×). The digested samples were analyzed by Western blotting using antibodies against RSF1 and GAPDH (loading control).

### Chromatin immunoprecipitation (ChIP)

ChIP was performed as described in our previous publication (Shi et al., 2022). ChIP was done with anti-RSF1 antibody (Abcam, ab109002, UK) and anti-IgG antibody (Abcam, ab171870, UK). Primers used for ChIP-qPCR are XIAP (forward, 5′- GAC​AGT​GCT​TAT​CTT​ACA​GGG​TTG-3′; reverse, 5′- GGA​ACC​GAG​AAG​CTG​ACC​TA-3′).

### Molecular docking

For molecular docking, the molecular structure of Tan I was obtained from the PubChem database (https://pubchem.ncbi.nlm.nih.gov/). The computed structure model of RSF1 (UniProt ID: Q96T23; AlphaFold entry: AF-Q96T23-F1-model_v4) was obtained from the RCSB Protein Data Bank (https://www.rcsb.org), which provides access to AlphaFold-predicted structural model. The AutoDock Vina (version 1.2.6) and Pymol software (version 2.4) were used for molecular docking. A blind docking strategy was employed to explore potential binding sites across the entire surface of RSF1, without prior assumptions regarding the binding pocket. The grid box was set to encompass the whole protein, with dimensions of 170 × 170 × 170 Å^3^ and a grid spacing of 1.0 Å.

### Statistical analysis

The SPSS 25.0 software was used for the statistical analyses. Data in this study were presented as mean with standard deviation (SD). Comparisons between groups were examined by Student’s t-test or analysis of variance test. The level of statistical difference was set at a *P* value (*P < 0.05, **P < 0.01 and ***P < 0.001).

## Results

### Tan I inhibits proliferation and colony formation in cervical cancer cells

**FIGURE 1:**
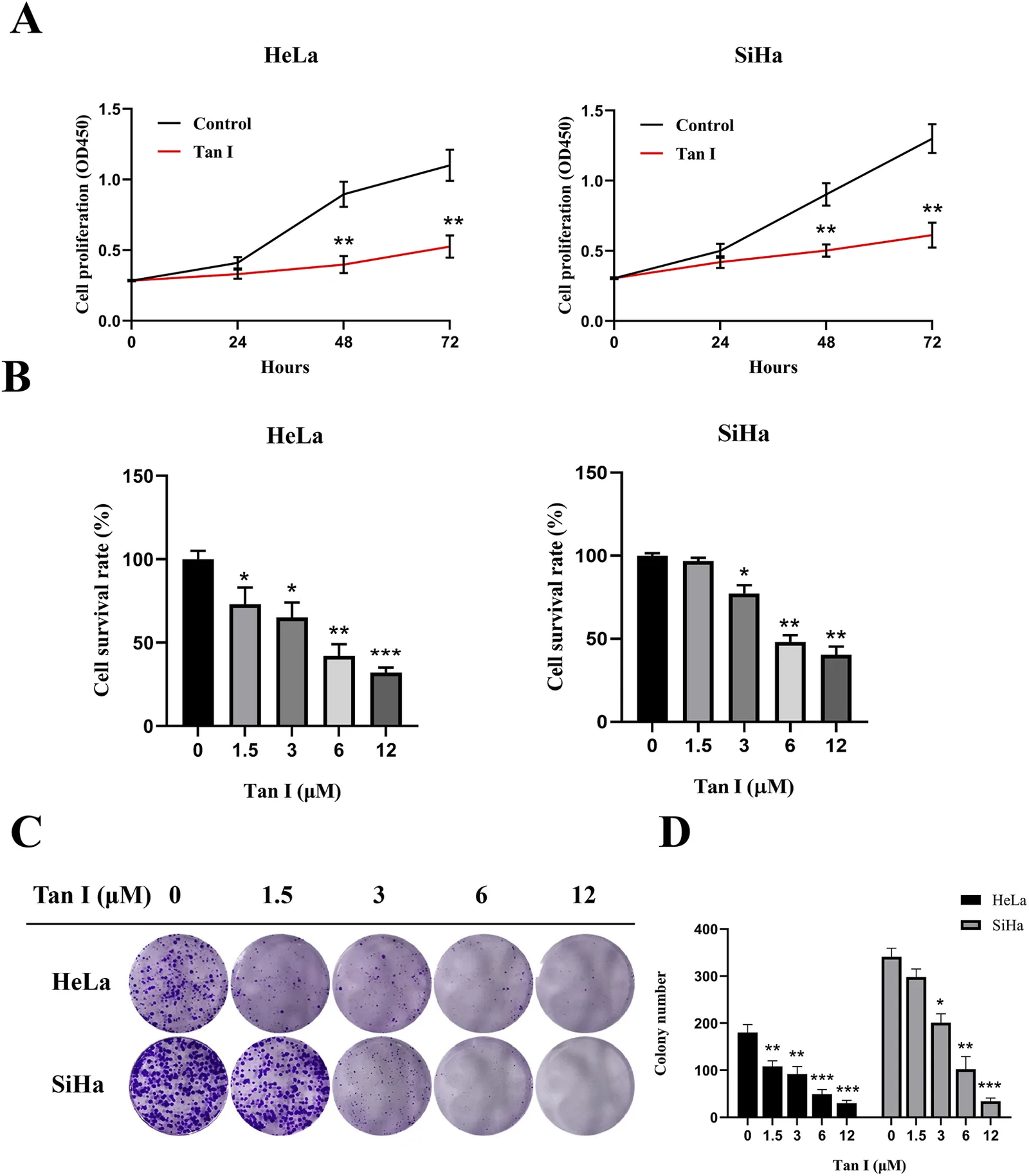
Tan I inhibits cell proliferation of cervical cancer cells. **(A)** The growth curve of HeLa and SiHa cells incubated with 6 μM Tan I for up to 72 h was measured by CCK-8 assays. DMSO-treated cells were considered as control. Error bars represent SD from three independent biological experiments. Student’s t-test, **P < 0.01. **(B)** The cell viability of HeLa and SiHa cells was evaluated following 48 h of treatment with increasing concentrations of Tan I (0, 1.5, 3, 6, and 12 μM). Error bars represent SD from three independent biological experiments. Student’s t-test, *P < 0.05, **P < 0.01, ***P < 0.001. **(C)** Tan I affected the colony formation of HeLa and SiHa cells. The cells were seeded in triplicate into 6-well plates and cultured for up to 12 days. The colonies were visualized by staining with crystal violet. **(D)** Quantitation of the colonies in colony formation. Error bars represent SD from three independent biological experiments. Student’s t-test, *P < 0.05, **P < 0.01, ***P < 0.001.

Based on prior literature, we initially selected 6 μM Tan I to evaluate its effects on cervical cancer cells under our experimental conditions. Consistent with previous reports (Cui et al., 2022), Tan I significantly suppressed the proliferation of HeLa and SiHa cells at 48 h, with the inhibitory effect becoming more pronounced over time (Figure 1A). To further assess Tan I’s cytotoxic effects, cell viability was measured using the CCK-8 assay following 48 h treatment with varying Tan I concentrations. As shown in Figure 1B, Tan I exhibited dose-dependent growth inhibition in both cell lines. Additionally, colony formation assays demonstrated that Tan I dose-dependently reduced the clonogenic capacity of cervical cancer cells (Figures 1C,D). Taken together, these findings indicate that Tan I significantly inhibits proliferation and colony formation in HeLa and SiHa cells.

### Tan I induces cell apoptosis in cervical cancer cells

**FIGURE 2:**
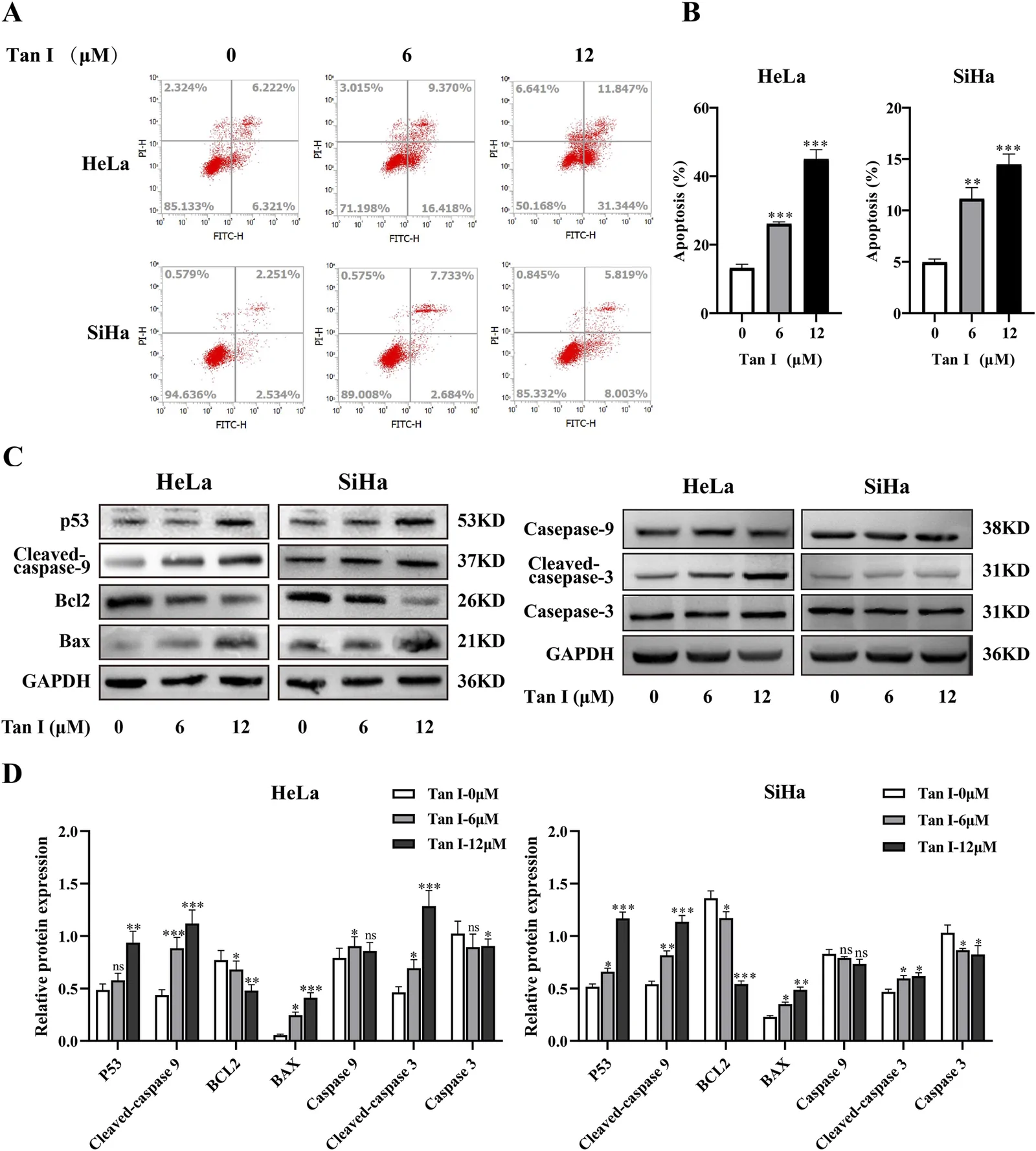
Tan I induces cervical cancer cell apoptosis. **(A)** Effect of the different concentrations of Tan I (0, 6, 12 μM) on the proportion of apoptotic cells after 48 h treatment. **(B)** Percentage of total apoptotic cells = Percentage of early apoptotic cells (Q3) + late apoptotic cells (Q2). Error bars represent SD from three independent biological experiments. Student’s t-test, **P < 0.01, ***P < 0.001. **(C)** Western blotting showed the effects of Tan I (0, 6, 12 μM) on Bcl-2, p53, Bax, Cleaved-Caspase-9, Caspase-9, Cleaved-Caspase-3 and Caspase-3 expression in HeLa and SiHa cells. **(D)** Quantitative analysis of Bcl-2, p53, Bax, Cleaved-Caspase-9, Caspase-9, Cleaved-Caspase-3 and Caspase-3 protein levels. Protein expression intensities were normalized to GAPDH. Error bars represent SD from three independent biological experiments. *P < 0.05, **P < 0.01, ***P < 0.001.

Apoptosis induction represents a critical mechanism underlying antitumor therapies. To further elucidate the inhibitory effects of Tan I on tumor cells, we evaluated its impact on apoptosis using flow cytometry in HeLa and SiHa cells. Compared with untreated controls, Tan I treatment significantly increased the proportion of apoptotic cells in a dose-dependent manner (Figures 2A,B). To further characterize Tan I-induced apoptosis, we analyzed the expression of key apoptosis-related proteins by Western blotting. Tan I treatment substantially downregulated the anti-apoptotic protein Bcl-2 while upregulating pro-apoptotic factors including Bax, cleaved caspase-9, and cleaved caspase-3 in both cell lines (Figures 2C,D). Increased p53 protein levels were also observed following Tan I treatment; however, given that HeLa and SiHa cells harbor HPV E6, which targets p53 for degradation, the functional significance of this increase is addressed in the Discussion. These findings suggest that Tan I triggers the intrinsic apoptotic pathway in cervical cancer cells.

### RSF1 as a candidate molecular target of tan I in cervical cancer cells

**FIGURE 3:**
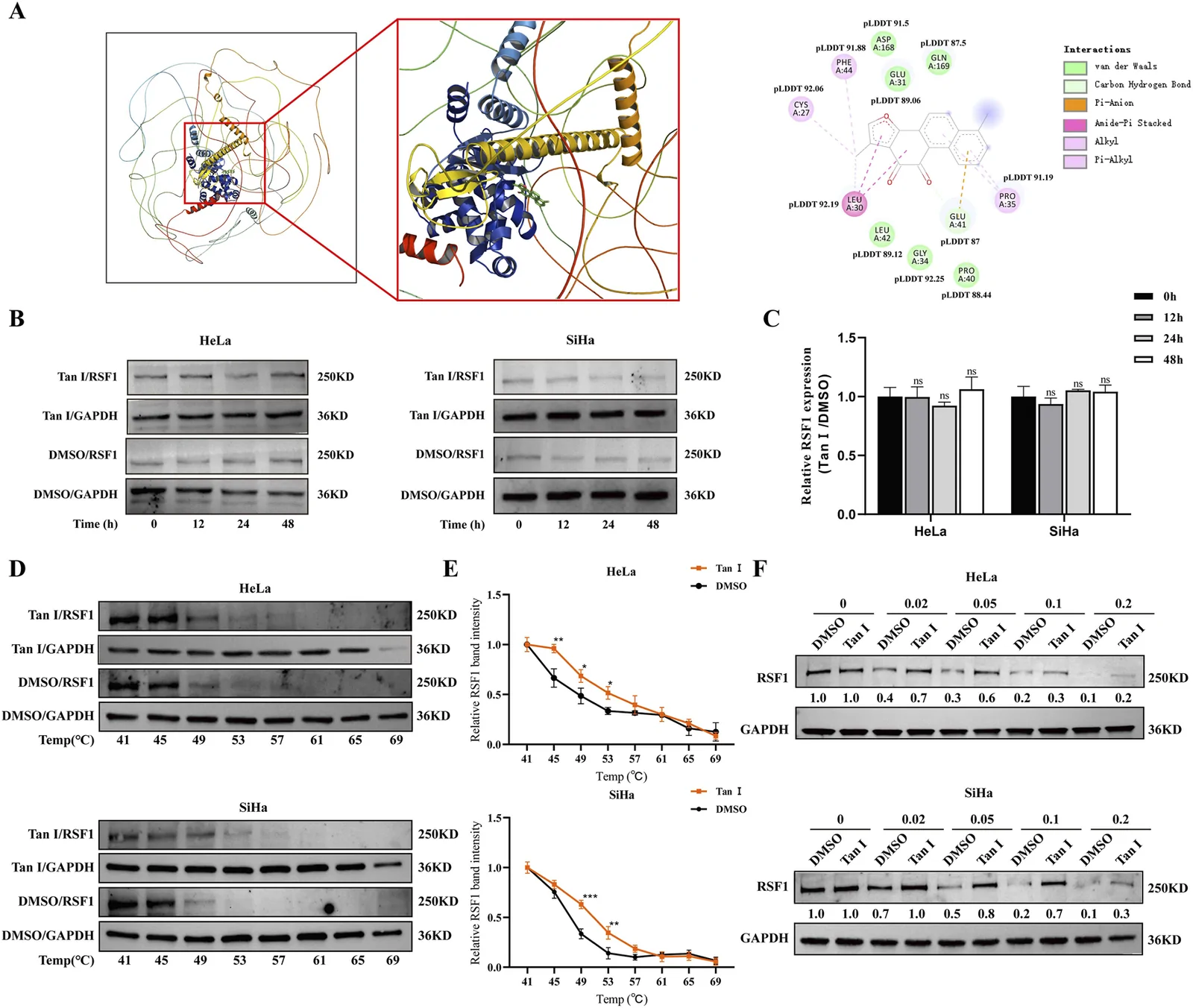
Tan I interacts with RSF1 in cervical cancer cells. **(A)** Molecular docking of Tan I with RSF1. Left: three-dimensional docking pose of Tan I (green sticks) within the predicted binding pocket of RSF1 (molecular surface). Right: two-dimensional schematic of the interactions between Tan I and surrounding residues. The predicted binding mode suggests that Tan I occupies a hydrophobic pocket on the RSF1 surface, with additional polar and van der Waals contacts contributing to the binding interface. Per-residue pLDDT scores of the key interacting residues are indicated in parentheses, with an average of 90.20. **(B)** Immunoblot analysis of RSF1 expression in cervical cancer cells treated with Tan I (6 μM) or DMSO for the indicated time periods (0–48 h). **(C)** Quantification of the immunoblots shown in **(B)**. The band intensities were measured and analyzed using ImageJ software, and RSF1 levels were normalized to GAPDH. All data are presented as mean ± SD from three independent experiments. **(D)** CETSA was performed to assess the engagement of Tan I with RSF1 in HeLa and SiHa cells. Cells were treated with Tan I (6 μM) or DMSO and subjected to the indicated temperature gradient (41°C–69 °C). The levels of soluble RSF1 were analyzed by Western blotting. **(E)** Quantification of the results in CETSA assay. The band intensities were measured using the ImageJ software and the relative band intensity was normalized to RSF1 protein level at 41 °C. Bars are means ± SD from three biological replicates under independent conditions. Student’s t-test, *P < 0.05, **P < 0.01, ***P < 0.001. **(F)** HeLa and SiHa cell lysates were incubated with Tan I (6 μM) for 1 h at room temperature and then subjected to proteolysis with increasing concentrations of pronase for 10 min. The pronase-resistant proteins in the presence or absence of Tan I were analyzed by Western blotting.

RSF1 was identified as a key cancer-driving gene in several cancer types, whose expression level was correlated with cancer progression and clinical outcomes (Cai et al., 2021). Molecular docking analysis predicted a strong binding affinity between Tan I and RSF1, with a binding energy of −8.90 kcal/mol (Figure 3A). Analysis of the binding mode revealed that Tan I engages with RSF1 through a network of non-covalent interactions, including hydrophobic stacking, electrostatic contacts, and weak hydrogen bonds. The calculated binding energy, which is substantially lower than the widely accepted threshold of −5.0 kcal/mol for significant interactions, suggesting a favorable binding mode. To assess the structural reliability of the docking model, we examined the per-residue pLDDT scores of the AlphaFold-predicted RSF1 structure. The residues at the Tan I–RSF1 binding interface exhibited pLDDT values ranging from 87.0 to 92.25, with an average of 90.20, indicating that this region is a well-folded, high-confidence domain. To determine whether Tan I affects RSF1 protein stability, we examined RSF1 protein levels by Western blot following Tan I treatment. As shown in Figures 3B,C, RSF1 protein levels remained largely unchanged compared to the vehicle control, indicating that Tan I does not induce RSF1 degradation. To investigate the potential interaction between Tan I and RSF1, we performed the cellular thermal shift assay (CETSA), in which drug-bound proteins stay in solution at high temperatures while unbound proteins denature and precipitate (Jafari et al., 2014). Our results showed that Tan I shifted the RSF1 heat denaturation curve to higher temperatures in HeLa and SiHa cells (Figures 3D,E), consistent with the possibility that Tan I engages with RSF1 in cervical cancer cells. To further support this observation, we performed a Drug Affinity Responsive Target Stability (DARTS) assay, which relies on the principle that ligand binding protects target proteins from protease digestion. As shown in Figure 3F, Tan I-treated lysates exhibited increased resistance to pronase digestion compared to the DMSO control. Taken together, these findings suggest RSF1 as a candidate molecular target of Tan I in cervical cancer cells.

### RSF1 contributes to the tumor progression of cervical cancer

**FIGURE 4:**
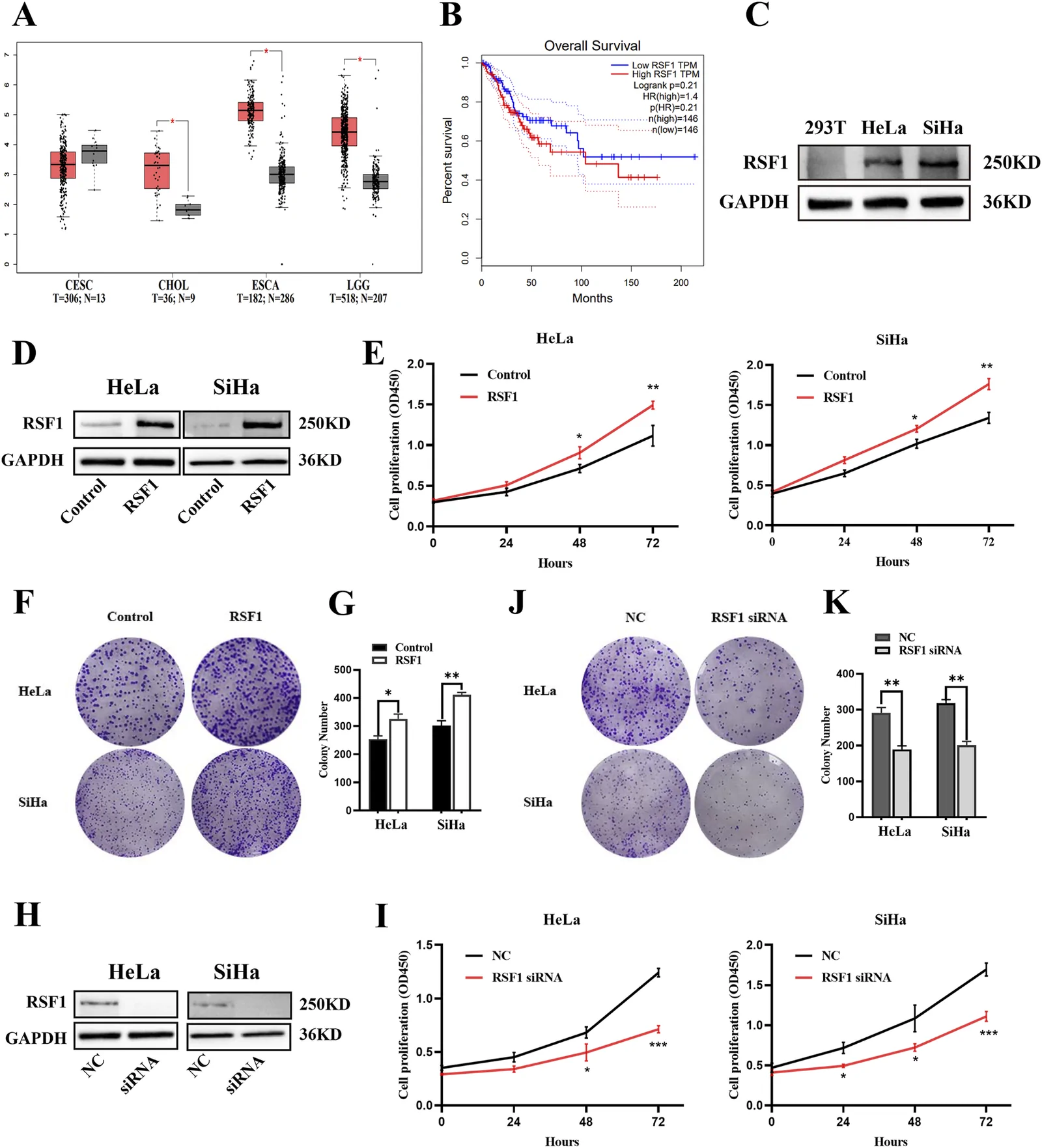
RSF1 mediates cell proliferation of cervical cancer cells. **(A)** The expression level of RSF1 in different tumors based on Gene Expression Profiling Interactive Analysis (GEPIA), matched with data from TCGA and GTEx databases. The red boxes represent tumor tissue, and the grey boxes represent normal tissue. **(B)** Effects of RSF1 expression level on the cervical cancer patient survival. The Kaplan-Meier survival curves revealed an association of overexpression RSF1 with shorter overall survival in cervical cancer patients based on GEPIA. **(C)** Western blotting detected RSF1 levels in HeLa and SiHa cells compared to the non-tumorigenic HEK293 T cells, and GAPDH as the internal reference. **(D)** Western blotting was performed to detect the RSF1 expression in cells transfected with RSF1 overexpression plasmid (RSF1) or empty vector (Control), respectively. **(E)** Overexpression of RSF1 promoted cell proliferation of HeLa and SiHa cells. The cell proliferation rates were monitored at the indicated times by CCK8 assay. Data shown are the average of three replicates. Student’s t-test, *P < 0.05, **P < 0.01. **(F)** The colony formation assay was performed in RSF1 overexpression and control cells. **(G)** Statistical results of colony formation in RSF1 overexpression and control cells are presented. Data shown are the average of three replicates. Student’s t-test, *P < 0.05, **P < 0.01. **(H)** Immunoblot was performed to detect the RSF1 expression in cells transfected with RSF1 siRNA or control (NC) siRNA. **(I)** The cell proliferation rates were monitored in RSF1 knockdown cells at the indicated times by CCK8 assay. Data shown are the average of three replicates and shown as mean ± SD. Student’s t-test, *P < 0.05, ***P < 0.001. **(J)** The colony formation assay was performed in RSF1 knockdown and control cells. **(K)** Statistical results of colony formation in RSF1 knockdown and control cells are presented. Data were obtained from three biological replicates. Student’s t-test, *P < 0.05, **P < 0.01.

Since previous studies have shown that RSF1 is strongly correlated with cancer development and affects clinical outcomes in a variety of tumor types (Cai et al., 2021; Wang et al., 2018), we next sought to study the roles of RSF1 in cervical cancer cells. According to GEPIA database (Gene Expression Profiling Interactive Analysis, http://gepia.cancer-pku.cn), RSF1 expression level was slightly lower in cervical squamous cell carcinoma and endocervical adenocarcinoma (CESC) compared to that in the normal controls, unlike its significantly higher expression in other tumor tissues such as cholangiocarcinoma (CHOL), esophageal carcinoma (ESCA), and brain Lower Grade Glioma (LGG) (Figure 4A). A Kaplan-Meier survival analysis showed that cervical cancer patients with high RSF1 levels had a shorter survival time than those patients with low RSF1 levels (Figure 4B), indicating that the level of RSF1 might be a useful predictor of prognosis for patients with cervical cancers. Western blotting showed that the expression levels of RSF1 were higher in HeLa and SiHa cells compared with the levels in non-tumorigenic HEK293 T cells (Figure 4C). To investigate the biological roles of RSF1 in cervical cancer cells, we transfected HeLa and SiHa cells with an expression vector encoding RSF1 (pLVX-RSF1). As demonstrated in Figure 4D, expressed pLVX-RSF1 cells had higher RSF1 protein levels than the control cells carrying the pLVX empty vector. CCK-8 and colony formation assays showed that upregulation of RSF1 promoted cell proliferation and increased the colony numbers in cervical cancer cells (Figures 4E–G). Conversely, we knocked down RSF1 expression by using specific RSF1 siRNA (Figure 4H). The results showed that downregulation of RSF1 suppressed cell growth and proliferation in cervical cancer cells (Figures 4I–K). According to the aforementioned studies, RSF1 may therefore function as a potential biomarker and therapeutic target for cervical cancer.

### The anti-proliferation effect of tan I in cervical cancer cells is dependent on RSF1

**FIGURE 5:**
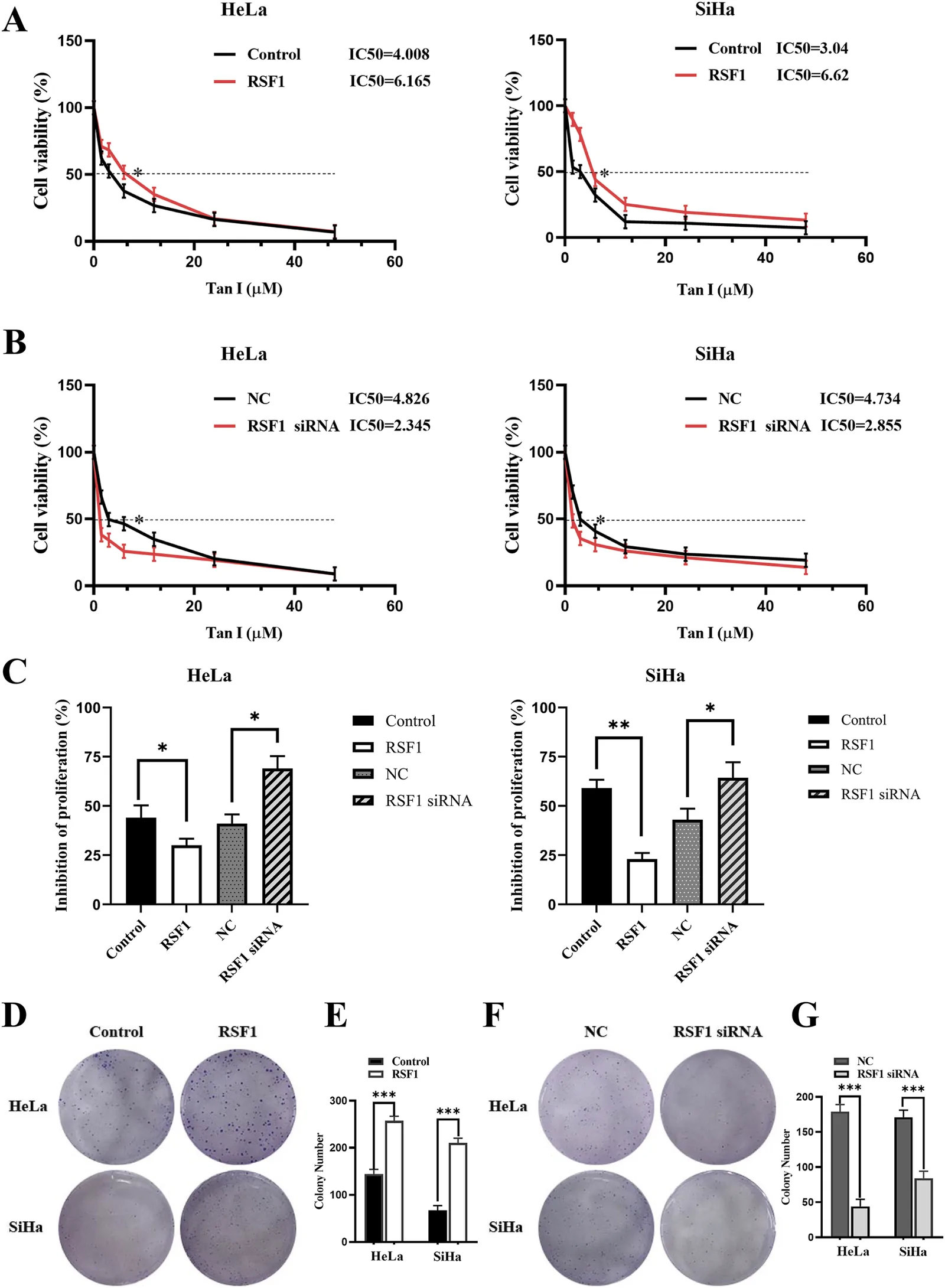
The inhibition effects of Tan I on the viability of cervical cancer cells depend on RSF1. **(A)** The inhibition effects of Tan I at various concentrations for 48 h on the growth of RSF1 overexpression cells and control cells. The dotted lines represent the corresponding concentration of IC_50_. Data shown are the average of three replicates. Student’s t-test, *P < 0.05. **(B)** Cell viability percentages in RSF1 knockdown cells and control cells treated with the different concentrations of Tan I for 48 h. The dotted lines represent the corresponding concentration of IC_50_. Data shown are the average of three replicates. Student’s t-test, *P < 0.05. **(C)** The inhibition of proliferation was tested by CCK-8 assay in HeLa and SiHa cells with overexpression of RSF1 after treatment with 6 μM Tan I for 48 h. Data were obtained from three biological replicates. Student’s t-test, *P < 0.05, **P < 0.01. **(D)** The colony formation assay was performed in RSF1 overexpression and control cells. The cells were incubated with 1.5 μM Tan I for 12 days and then stained with crystal violet. **(E)** Statistical results of colony formation RSF1 overexpression and control cells are presented. Data were obtained from three biological replicates and shown as mean ± SD. *P* values were determined by Student’s t-test, ***P < 0.001. **(F)** The colony formation assay was performed in RSF1 knockdown and control cells incubated with 1.5 μM Tan I for 12 days. **(G)** Statistical results of colony formation in RSF1 knockdown and control cells are presented. Data were obtained from three biological replicates. *P* values were determined by Student’s t-test, ***P < 0.001.

Based on the above results that RSF1 was involved in cancer cell development and RSF1 was a molecular target of Tan I, we speculated that Tan I might inhibit cervical cancer cell growth via modulating RSF1. To know whether RSF1 affects Tan I sensitivity in cervical cancer cells, the IC_50_ values of Tan I treatment were measured in cervical cancer cells with upregulation or downregulation of RSF1. As shown in Figure 5A, the IC_50_ values of Tan I in control HeLa and SiHa cells (expressed empty pLVX vector) were 4.008 μM and 3.040 μM, respectively, while overexpression of RSF1 increased the IC_50_ values of Tan I in HeLa and SiHa cells to 6.165 μM and 6.620 μM, respectively. On the contrary, knockdown of RSF1 by siRNA induced a significant decrease in the IC_50_ values in HeLa and SiHa cells (Figure 5B). In addition, overexpression of RSF1 led to a substantial reduction in the inhibitory effect of Tan I on cervical cancer cells, whereas knockdown of RSF1 induced an increase of the inhibitory effect of Tan I in HeLa and SiHa cells (Figure 5C), suggesting that the expression of RSF1 affects the inhibition effect of Tan I on the cervical cancer cells. The similar results were observed in colony formation assay that overexpression of RSF1 impaired the sensitivity of cervical cancer cells to Tan I, whereas knockdown of RSF1 improved the sensitivity (Figures 5D–G). These results indicated that RSF1 acts as a pro-survival factor, and that Tan I exerts its effects, at least in part, by counteracting this protective function.

### Tan I inhibits cervical cancer cell proliferation through RSF1/NF-κb pathway

**FIGURE 6:**
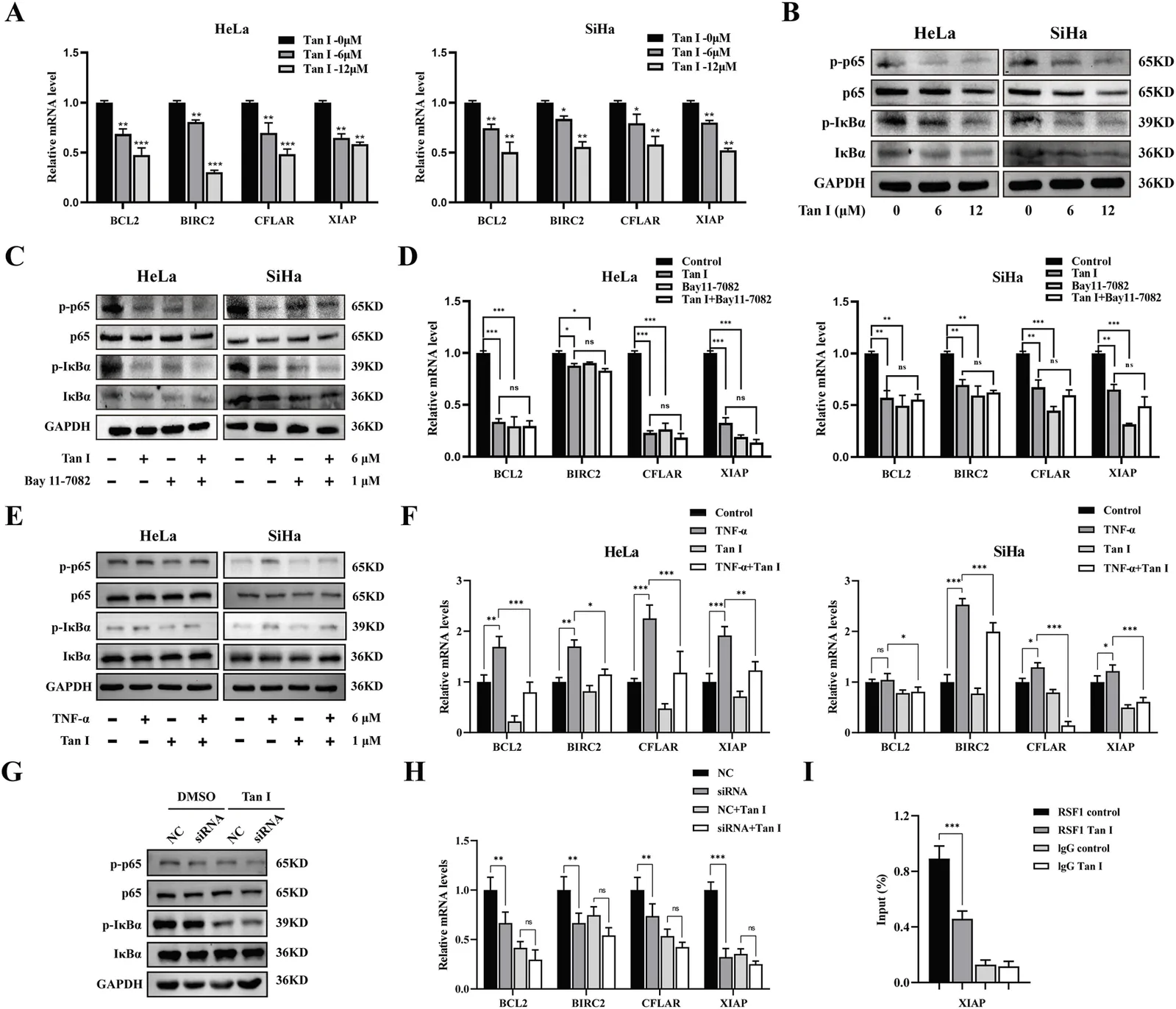
Tan I suppressed NF-κB pathway in cervical cancer cells. **(A)** RT-qPCR analysis of the transcript levels of *BCL2*, *BIRC2*, *CFLAR*, and *XIAP* genes in HeLa and SiHa cells with different concentrations (0, 6, 12 μM) of Tan I for 48 h. Data were obtained from three biological replicates. *P* values were determined by Student’s t-test, *P < 0.05, **P < 0.01, ***P < 0.001. **(B)** The protein levels of p-p65, p65, p-IκBα, and IκBα were analyzed by Western blotting with specific antibodies. GAPDH was used as a loading control. **(C)** Immunoblot analysis was performed using specific antibodies in cervical cancer cells in the presence or absence of Tan I (6 μM) in combination with the presence or absence of Bay 11–7082 (1 μM). **(D)** The mRNA levels of *BCL2*, *BIRC2*, *CFLAR*, and *XIAP* genes in cervical cancer cells with 6 μM Tan I plus 1 μM Bay 11–7082 treatment for 48 h were detected by RT-qPCR. The data were representative of three different experiments. *P* values were determined by Student’s t-test, *P < 0.05, **P < 0.01, ***P < 0.001. **(E)** Western blotting analysis of p-p65, p65, p-IκBα, and IκBα levels in cervical cancer cells with TNF-α (10 ng/mL), Tan I (6 μM), or their combination. GAPDH was used as a loading control. **(F)** RT-qPCR analysis of *BCL2*, *BIRC2*, *CFLAR*, and *XIAP* mRNA levels in cervical cancer cells with TNF-α (10 ng/mL) with or without 6 μM Tan I. The data were representative of three different experiments. *P* values were determined by Student’s t-test, *P < 0.05, **P < 0.01, ***P < 0.001. **(G)** Western blotting analysis of p-p65, p65, p-IκBα, and IκBα levels in RSF1-knockdown HeLa cells with Tan I (6 μM) for 48 h. Control cells were transfected with non-targeting siRNA. GAPDH was used as the internal reference. **(H)** The mRNA levels of NF-κB-induced genes were examined in RSF1-knockdown HeLa cells treated with 6 μM Tan I or DMSO. Data are presented as mean ± SD from three independent experiments. **(I)** Chromatin immunoprecipitation (ChIP) analysis of RSF1 occupancy at the XIAP promoter in HeLa cells treated with Tan I (6 μM) or DMSO. ChIP was performed using anti-RSF1 antibody or control IgG. Data are presented as means ± SD from three biological replicates. *P < 0.05, **P < 0.01, ***P < 0.001.

Previous studies have shown that RSF1 contributes to the cell proliferation and chemoresistance of tumor cells by regulating NF-κB signaling pathway (Liu et al., 2017; Yang et al., 2014). We then hypothesize that Tan I exerts tumor-suppressive effects through modulating RSF1/NF-κB pathway. To investigate the underlying mechanism of Tan I-inhibited proliferation, the transcript levels of NF-κB-dependent genes were detected following exposure to Tan I in HeLa and SiHa cells. As shown in Figure 6A, the expression levels of *BCL2*, *BIRC2*, *CFLAR*, and *XIAP* genes were markedly decreased by exposure to Tan I in HeLa and SiHa cells compared with those control group. The results suggested that Tan I treatment suppressed the transcripts of NF-κB-targeted genes in a dose-dependent manner in the cervical cancer cells. In addition, the protein levels of NF-κB were detected with Tan I treatment in HeLa and SiHa cells. Western blotting showed that the treatment of Tan I significantly decreased the protein levels of p-p65 and p-IκBα in HeLa and SiHa cells (Figure 6B), indicating that Tan I impaired the activity of NF-κB in the cervical cancer cells. To corroborate this, Bay 11–7082, an NF-κB phosphorylation inhibitor, was used to further verify the effect of Tan I on the inhibition of NF-κB signaling pathway in cervical cancer cells. The results showed that the protein levels of p-p65 and p-IκBα were significantly inhibited in Bay 11–7082 treated cervical cancer cells (Figure 6C). However, when HeLa and SiHa cells were co-treated with Tan I and Bay 11–7082, the inhibitory levels of NF-κB pathway proteins were no further higher compared with the cells that were treated by Tan I or Bay 11–7082 alone (Figure 6C). Similarly, the mRNA levels of NF-κB-dependent genes were downregulated by Tan I or Bay 11–7082 individually, but their combination did not produce an additional decrease (Figure 6D). To further validate these observations, we used TNF-α, a well-known NF-κB activator. HeLa and SiHa cells were pretreated with TNF-α (10 ng/mL) followed by Tan I exposure. As shown in Figure 6E, TNF-α alone increased p65 and IκBα phosphorylation, whereas subsequent Tan I treatment markedly attenuated this effect. Consistently, TNF-α induced the transcription of *BCL2*, *BIRC2*, *CFLAR*, and *XIAP*, and Tan I reversed this upregulation (Figure 6F). We also examined NF-κB activity in RSF1 siRNA-treated HeLa cells. The results showed that RSF1 knockdown reduced p-p65 and p-IκBα levels, as well as the transcript levels of *BCL2*, *BIRC2*, *CFLAR*, and *XIAP* (Figures 6G,H). Moreover, Tan I treatment did not cause obvious further inhibition of NF-κB activity in RSF1-silenced cells. Previous research has shown that RSF1 regulates NF-κB-induced gene expression by binding to the XIAP promoter region in ovarian cancer cells (Yang et al., 2014). Accordingly, our ChIP assay revealed that Tan I treatment reduced RSF1 occupancy at the XIAP promoter in HeLa cells (Figure 6I). Collectively, these findings indicate that Tan I inhibits cervical cancer cell proliferation, at least in part, through modulation of the RSF1/NF-κB axis.

### Combination of Tan I and carboplatin reveals enhanced inhibition of cervical cancer cell growth

**FIGURE 7:**
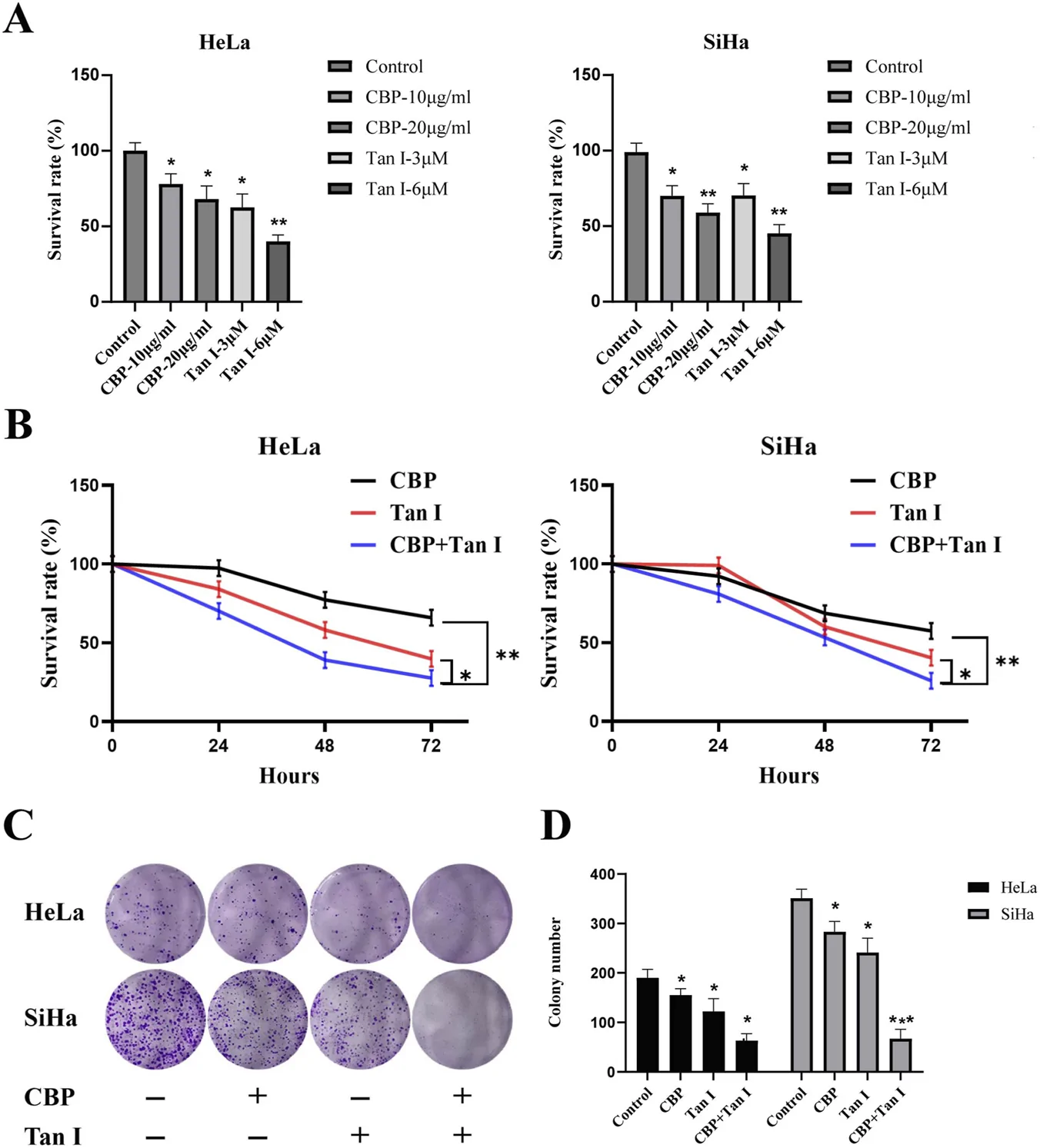
The combination of Tan I and carboplatin enhanced inhibition effects on the growth of cervical cancer cells. **(A)** Inhibition effects of carboplatin (CBP) and Tan I on the growth of cervical cancer cells individually. Cell survival rates in HeLa and SiHa cells were monitored by CCK-8 assay after treatment with CBP (10 μg/mL, 20 μg/mL) or Tan I (3μM, 6 μM) for 48 h. **(B)** Effects of CBP combined with Tan I on the growth of cervical cancer cells. After treatment with CBP (10 μg/mL), Tan I (3 µM), or the same amount of CBP plus Tan I for the indicated time, the cell viability percentages were detected by CCK-8 assays. Data were representative of three different experiments and presented as the mean ± SD. *P < 0.05, **P < 0.01 b y ANOVA test. **(C)** After treating with CBP (10 μg/mL), Tan I (3 µM), or the same amount of CBP plus Tan I in HeLa and SiHa cells for 12 days, the colonies were stained with crystal violet, and colony numbers were counted. **(D)** Statistical results of colony formation in **(C)** are presented. The data were obtained from three independent biological replicates, Student’s t-test, *P < 0.05, **P < 0.01, ***P < 0.001.

Since certain components of medicinal herb have been proven to improve the efficacy of chemotherapy in many types of tumors, we then investigate the combination of Tan I with conventional anti-tumor drugs for their effect on the proliferation of cervical cancer cells. Carboplatin (CBP) is one of the most active cytotoxic agents in the clinical treatment of cervical cancer (Ho et al., 2016). Firstly, we examined the effect of CBP (10 μg/mL and 20 μg/mL) and Tan I (3 μM and 6 μM) on the growth inhibition of cervical cancer cells. Treatment of CBP (10 μg/mL) or Tan I (3 μM) alone for 48 h effectively suppressed the cell proliferation of cervical cancer cells (Figure 7A). Next, we studied the effect of Tan I in combination with CBP. The CCK-8 assay showed that combined treatment with Tan I and CBP produced significantly greater inhibition than that with either agent alone at the same concentration (3 μM Tan I or 10 μg/mL CBP) (Figure 7B). In accordance with the CCK-8 results, colony formation assays showed that the combination of Tan I (3 μM) and CBP (10 μg/mL) enhanced the growth inhibition on cervical cancer cells (Figures 7C,D). Therefore, Tan I improved the anti-tumor activity of carboplatin in cervical cancer and Tan I has potential for use as an adjuvant agent combined with antitumor drugs.

## Discussion

Tan I is a diterpene quinone compound with a variety of pharmacological effects, including protecting the cardiovascular system, anti-inflammatory properties, neuroprotective effects, antioxidant stress, and anticancer effects. Its broad therapeutic potential is attributed to its ability to modulate multiple pathways/targets (Ke et al., 2023). Accumulating evidence has demonstrated that Tan I exerts antitumor activity across various cancers, including osteosarcoma (Ye et al., 2022), colorectal cancer (Lu et al., 2016), hepatoma (Liu and Liu, 2020), ovarian cancer (Zhou et al., 2020), breast cancer (Jin et al., 2022), and prostate cancer (Gong et al., 2011). In this study, we investigated the effects of Tan I on cervical cancer cells. Our results showed that Tan I inhibited the proliferation of HeLa and SiHa cells in a dose-dependent manner, as determined by cell proliferation and colony formation assays. Furthermore, Tan I significantly induced cell apoptosis in both cell lines, consistent with the previous reports that Tan I suppresses metastasis in human cervical cancer cells (Cui et al., 2022; Dun and Gao, 2019). Our data demonstrate that Tan I activates the intrinsic apoptotic pathway, as evidenced by increased cleavage of caspase-9 and the downstream effector caspase-3, without altering the expression levels of the full-length proteins. In addition, Tan I upregulates p53 and Bax protein levels, while downregulating the levels of Bcl2 in cervical cancer cells (Figure 2). However, given that HeLa and SiHa cells harbor HPV E6, which targets p53 for ubiquitin-mediated degradation, and that we did not assess p53 target gene activation, we cannot conclude that p53 is functionally activated in this context. Instead, our findings support a p53-independent apoptotic mechanism, in which RSF1 serves as a candidate molecular target of Tan I. By suppressing NF-κB activity and subsequently downregulating anti-apoptotic genes such as *XIAP*, *CFLAR*, and *BCL2*, Tan I counteracts the protective function of RSF1, thereby triggering apoptosis through activation of the caspase cascade (caspase-9 and caspase-3). Collectively, these data suggest that Tan I-induced apoptosis in cervical cancer cells is mediated through the RSF1/NF-κB axis in a p53-independent manner.

RSF1 is an essential interphase centromere-associated protein that mainly functions in maintaining chromosomal stability and executing DNA repair at a suitable level. However, RSF1 is often overexpressed in many types of cancers, related to anticancer drug resistance and lower overall survival rates (Cai et al., 2021). In our analysis using the TCGA and GTEx databases, RSF1 is overexpressed in cholangiocarcinoma (CHOL), esophageal carcinoma (ESCA), and brain lower-grade glioma (LGG) but not in cervical squamous cell carcinoma (CESC) tumor tissues compared with the normal tissues (Figure 4A). However, another study using the ONCOMINE database showed that the expression of RSF1 was significantly higher in cervical cancer tissues than in normal tissues (Tian et al., 2020). Similarly, another immunohistochemical study reported that RSF1 was significantly higher expressed in 160 malignant cervical carcinoma sections than that in 20 normal tissue sections (Wang et al., 2018). The discrepancy between TCGA-based analysis and these published observations may be attributable to several factors. First, differences in database composition, sample size, and normalization methods between TCGA/GTEx and ONCOMINE could contribute to the divergent results. Second, the histological and genetic heterogeneity of cervical cancer specimens, varying in HPV type, tumor stage, and molecular subtype, may influence RSF1 expression levels, potentially leading to inconsistent findings across different patient cohorts. Third, RSF1 upregulation may occur in specific cervical cancer subtypes rather than uniformly across all cases, which could be masked in pooled TCGA analyses but captured in smaller, more selected cohorts. Despite the inconsistency in expression levels across datasets, Kaplan-Meier survival analysis of TCGA data revealed that cervical cancer patients with high RSF1 expression had significantly shorter overall survival than those with low RSF1 expression (Figure 4B), suggesting that RSF1 may serve as a useful prognostic predictor for cervical cancer patients. This finding underscores the clinical relevance of RSF1 in cervical cancer, independent of whether its upregulation is uniformly detected at the transcriptomic level in bulk tumor analyses. Given these considerations, we further examined RSF1 protein levels in cervical cancer cell lines. Our *in vitro* data demonstrated that RSF1 was elevated in HeLa and SiHa cells compared with non-tumorigenic HEK293 T cells (Figure 4C). Nevertheless, we acknowledge that HEK293T cells, being embryonic kidney-derived, do not represent an ideal normal control for cervical epithelium. Future studies using immortalized normal cervical epithelial cell lines (such as End1/E6E7 or ECT1/E6E7) or primary ectocervical epithelial cells would provide a more appropriate comparison and strengthen the pathological relevance of our findings. Despite this limitation, the consistent overexpression of RSF1 in two independent cervical cancer cell lines, together with the prognostic significance from survival analysis and prior literature support, suggests that RSF1 confers a survival advantage to cervical cancer cells.

To identify the molecular target of Tan I in cervical cancer cells, we focused on RSF1 based on its established role in NF-κB regulation and cancer cell survival. This candidate-driven approach was further validated by CETSA and DARTS assays, which supported the engagement of Tan I with RSF1 (Figure 3). While these biophysical assays collectively support an interaction between Tan I and RSF1, we acknowledge that they do not formally establish direct physical binding with a defined affinity, which would require additional approaches such as surface plasmon resonance or isothermal titration calorimetry. Nevertheless, the consistent results across these orthogonal assays strengthen the candidacy of RSF1 as a molecular target of Tan I. RSF1 has been reported to activate NF-κB signaling pathway via upregulation of p-IκB and NF-κB p65 protein in various cancer cells (He et al., 2019; Zhao and Hong, 2021). The NF-κB signaling pathway is frequently activated in cervical cancer and plays crucial roles in regulating cell cycle, apoptosis, tumorigenesis, and resistance to chemotherapy (Deng et al., 2024). In this study, we found that Tan I treatment noticeably decreased the expression of p-p65 and p-IκB, as well as the transcriptional expression of NF-κB downstream target genes in cervical cancer cells (Figures 6A,B). Moreover, co-treatment with NF-κB inhibitor Bay 11–7082 and Tan I did not result in enhanced suppression of NF-κB activity (Figures 6C,D), whereas Tan I partially reversed the activating effect of NF-κB agonist TNF-α (Figures 6E,F). In addition, our ChIP assay revealed that Tan I treatment reduced RSF1 occupancy at the XIAP promoter (Figure 6I), suggesting that Tan I may interfere with the physical association of RSF1 with its target genes. Collectively, we propose that Tan I inhibits the pro-survival function of RSF1 in cervical cancer cells by impairing its ability to associate with chromatin, rather than by promoting its degradation. Nevertheless, we acknowledge that Tan I, as a diterpene quinone with diverse bioactivities, may interact with multiple targets in cancer cells. Although our candidate-based approach identified RSF1 as a key mediator, unbiased target identification, such as pull-down coupled with mass spectrometry, would provide a more comprehensive mechanistic understanding and will be pursued in our future studies.

The combination of natural products with conventional anticancer agents has received increasing attention in recent years (Cao, 2024). There is accumulating evidence that natural products can enhance the efficacy of chemotherapeutic drugs and help circumvent drug resistance (Cheon and Ko, 2022). Carboplatin is one of the most widely used chemotherapeutic agents in the clinical management of cervical cancer (Ho et al., 2016). In this study, we evaluated the combined effect of Tan I and carboplatin on cervical cancer cells and observed enhanced anticancer efficacy (Figure 7), indicating that the combination of Tan I and carboplatin may serve as a promising therapeutic strategy for cervical cancer. It should be noted, however, that the IC_50_ values of Tan I in our *in vitro* assays ranged from approximately two–7 μM, which are higher than the plasma concentrations typically achievable following oral administration of the free compound in animal models. Nevertheless, various nanodelivery strategies, including co-delivery liposomes and self-emulsifying drug delivery systems, have been shown to significantly improve the bioavailability of Tan I. Future studies employing such optimized formulations are warranted to further evaluate the *in vivo* efficacy and safety of Tan I in preclinical cervical cancer models. Additionally, our findings are primarily derived from two HPV-positive cervical cancer cell lines (HeLa and SiHa); future studies using HPV-negative lines (e.g., C33 A) and a broader panel of cervical cancer cells are warranted to determine whether the observed Tan I–RSF1–NF-κB regulatory axis is broadly applicable across different cervical cancer subtypes.

## Conclusion

Taken together, the present study illustrated that Tan I inhibited proliferation and induced apoptosis in cervical cancer cells. Mechanistically, our findings suggest that Tan I may interact with RSF1, thereby suppressing the NF-κB signaling pathway and downregulating key anti-apoptotic genes, including *XIAP*, *CFLAR*, *BCL2*, and *BIRC2*. In addition, we observed that Tan I enhanced the anticancer activity of carboplatin in cervical cancer cells. Therefore, Tan I may represent a novel candidate for the treatment of cervical cancer, warranting further preclinical evaluation.

## Data Availability

The original contributions presented in the study are included in the article/Supplementary Material, further inquiries can be directed to the corresponding authors.
